# Corrections: “Synthesis of Sulfonamides and Evaluation of Their Histone Deacetylase (HDAC) Activity” by Jung *et al*., *Molecules* 2007, *12*, 1125-1135 

**DOI:** 10.3390/molecules14051950

**Published:** 2009-05-25

**Authors:** Derek J. McPhee

**Affiliations:** Editor-in-Chief, *Molecules*. Molecular Diversity Preservation International (MDPI), Kandererstrasse 25, Basel CH-4057, Switzerland; E-mail: mcphee@mdpi.org

At the request of the authors of this paper [1], we wish to announce the following corrections:

The list of authors and their institutional affiliations is revised to:

Seikwan Oh ^1^, Hyung–In Moon ^2^, Il–Hong Son ^2^, Jae–Chul Jung ^1,* ^and Mitchell A. Avery ^3^

^1^ Department of Neuroscience and Medical Research Institute, College of Medicine, Ewha Womans University, Seoul 158–710, Korea

^2^ Department of Neuroscience and Inam Neuro Science Research Center, Wonkwang University Sanbon Medical Center, Sanbondong, Gunpocity, Kyunggido, 435-040, Korea; E-mails: himoon@wonkwang.ac.kr (Hyung–In Moon); soniag@wonkwang.ac.kr (Il–Hong Son)

^3^ Department of Medicinal Chemistry, School of Pharmacy, National Center for Natural Products Research, & Department of Chemistry, University of Mississippi, University, MS 38677-1848, USA; E-mail: mavery@olemiss.edu.

* To whom correspondence should be addressed; E-mail: jcjung@ewha.ac.kr

The Acknowledgements section is revised to: 

This work has been supported by the KOSEF Brain Neurobiology Grant (2006), the Ewha Global Challenge grant (BK21) and Cooperative Agreement Number 1-U01 C1000211 from the U.S. Centers for Disease Control and Prevention (M.A.A.). 

This Errata note will be linked henceforth to the original paper. The corresponding author and other coauthors wish to apologize to Dr Avery, the University of Mississippi and the C.D.C. for the omission of their contributions to this research, and to the readership of *Molecules* for any inconvenience caused. 
